# Time-Series Analysis of Mortality Effects of Fine Particulate Matter Components in Detroit and Seattle

**DOI:** 10.1289/ehp.1002613

**Published:** 2010-12-30

**Authors:** Jiang Zhou, Kazuhiko Ito, Ramona Lall, Morton Lippmann, George Thurston

**Affiliations:** New York University School of Medicine, Tuxedo, New York, USA

**Keywords:** cardiovascular mortality, chemical components, distributed lag model, gaseous pollutants, PM_2.5_, respiratory mortality, time-series analysis

## Abstract

**Background:**

Recent toxicological and epidemiological studies have shown associations between particulate matter (PM) and adverse health effects, but which PM components are most influential is less well known.

**Objectives:**

In this study, we used time-series analyses to determine the associations between daily fine PM [PM ≤ 2.5 μm in aerodynamic diameter (PM_2.5_)] concentrations and daily mortality in two U.S. cities—Seattle, Washington, and Detroit, Michigan.

**Methods:**

We obtained daily PM_2.5_ filters for the years of 2002–2004 and analyzed trace elements using X-ray fluorescence and black carbon using light reflectance as a surrogate measure of elemental carbon. We used Poisson regression and distributed lag models to estimate excess deaths for all causes and for cardiovascular and respiratory diseases adjusting for time-varying covariates. We computed the excess risks for interquartile range increases of each pollutant at lags of 0 through 3 days for both warm and cold seasons.

**Results:**

The cardiovascular and respiratory mortality series exhibited different source and seasonal patterns in each city. The PM_2.5_ components and gaseous pollutants associated with mortality in Detroit were most associated with warm season secondary aerosols and traffic markers. In Seattle, the component species most closely associated with mortality included those for cold season traffic and other combustion sources, such as residual oil and wood burning.

**Conclusions:**

The effects of PM_2.5_ on daily mortality vary with source, season, and locale, consistent with the hypothesis that PM composition has an appreciable influence on the health effects attributable to PM.

Over the past few decades, there has been growing interest in the adverse health effects of ambient air particulate matter (PM) on humans. Evidence from epidemiological studies has accumulated to support associations between PM and adverse health effects (e.g., Dominici et al. 2006; Peng et al. 2005; Schwartz and Morris 1995; Zanobetti and Schwartz 2009). A number of recent multicity time-series studies have demonstrated associations between daily mortality and the mass concentration of PM_2.5_ (PM ≤ 2.5 μm in aerodynamic diameter) (e.g., Laden et al. 2000; Ostro et al. 2006).

There is also growing evidence that PM_2.5_ mass alone might not be able to explain the health outcomes (Franklin et al. 2008; Lippmann et al. 2006; Sarnat et al. 2008), because ambient PM_2.5_ is chemically nonspecific and consists of various components and compounds [trace elements, elemental carbon (EC), organic carbon, and sulfate], and the toxicity of each of these chemical components and their mixtures may vary. These different components originate from various sources, such as traffic-related emissions, biomass combustion, residual oil burning, and resuspended dust. Metals that are components of ambient PM_2.5_, and especially the transition metals, have been cited as likely toxic components. The focus has often been on iron (Fe), vanadium (V), nickel (Ni), chromium, copper, and zinc (Zn) based on their ability to generate reactive oxygen species in biological tissues. The National Research Council (NRC) has highlighted the importance of investigating characteristics and chemical components of PM that contribute to their toxicity (NRC 2004). Focusing regulations on the most toxic PM components could protect public health more effectively and at a lower cost.

The Chemical Speciation Network (CSN) initiated by the U.S. Environmental Protection Agency (EPA) in 2000 has provided new opportunities for studies on PM components’ health effects (U.S. EPA 2010b). However, the sampling frequency of only 1-in-3 or 1-in-6 days severely limits the statistical power for time-series analysis. To date, because of this statistical power issue, most studies have used annual or seasonal averages of the PM_2.5_ in second-stage regression to explain the city-to-city differences in PM health effect estimates obtained in the first-stage time-series analysis in multiple individual cities (Bell et al. 2009; Franklin et al. 2008; Lippmann et al. 2006; Zanobetti et al. 2009). Direct regressions of chemical species in time-series models were limited either to a few chemical components that explained a substantial amount of PM_2.5_ mass (Peng et al. 2009) or to the analysis of multiple cities using earlier data. These analyses produced wide confidence bands, an apparent reflection of the issue of small sample size (Ostro et al. 2007). In the present study, we analyzed 3 years of daily speciation data in two U.S. cities that allowed an examination of the role of chemical species in PM_2.5_-associated health effects with larger sample sizes than those relying on CSN data.

## Materials and Methods

Detroit’s air quality is heavily influenced by a variety of industries, including motor vehicle factories, refinery operations, power plants, and metalworking plants. Accordingly, a number of air pollution–health effect studies have been conducted in Detroit (Harkema et al. 2004; Ito 2003; Morishita et al. 2004; Schwartz 1991; Schwartz and Morris 1995). Seattle, a major seaport for commerce with Asia, has container shipping, as well as warehousing facilities that are located northwest and west of the monitoring site, and the Port of Seattle contributes the largest source of oil combustion effluents in Seattle (Kim et al. 2004; Maykut et al. 2003). Seattle also has some other localized PM sources, such as residential wood burning. These two cities also have large populations, different climates, and different pollution mixes.

### PM_2.5_ cumulative filter samples

Two sets of 24-hr PM_2.5_ samples for the 3-year period 2002 through 2004 were collected on 47-mm Teflon filters using samplers operating at a flow rate of 16.7 L/min. These filters were from two monitoring sites: Detroit, Michigan (Allen Park, site ID 26-163-0001), and Seattle, Washington (Beacon Hill, site ID 53-033-0080) and were weighed pre- and postsampling to determine daily fine particle mass concentration. We obtained these filters from the Washington Department of Ecology (Seattle data) and Department of Environmental Quality (Detroit data) and subsequently sent them to the Research Triangle Institute (Research Triangle Park, NC) for analysis of trace element concentrations using energy-dispersive X-ray fluorescence (XRF). Black carbon, as a surrogate index of EC in the filter samples, was determined via reflectometry at New York University Department of Environmental Medicine (Tuxedo, NY).

### Choice of PM_2.5_ chemical components for data analysis

From the XRF analysis of 48 elements, we chose 10 chemical components for the health effects analysis based on toxicological consideration: aluminum (Al), Fe, potassium (K), sodium (Na), Ni, sulfur (S), silicon (Si), V, Zn, and EC. Source types were selected *a priori*, based on previous studies (Bell et al. 2009; Cooper and Watson 1980; Franklin et al. 2008; Gildemeister et al. 2007; Lippmann et al. 2006; Maykut et al. 2003; Thurston and Spengler 1985), as well as our cursory factor analysis (data not shown). Collectively, these 10 components represent the major emission sources in these two cities: Al and Si for soil, Fe and Zn for smelter effluents, Ni and V for residual oil burning, S for coal burning, EC for traffic, Na for sea salt, and K for wood burning. We removed several extreme values of K from the data set (from samples collected around 4 July 2002from samples collected around 4 July 2003, and 2004 that were influenced by fireworks).

### Gaseous pollutants data

We obtained daily concentrations of carbon monoxide (CO), ozone (O_3_), and nitrogen dioxide (NO_2_) from the Health Effects Institute (HEI) Air Quality Database (2010) and included these gaseous criteria pollutants into our models as potential confounders of PM components and to estimate their potential effects.

### Mortality data

We obtained National Center for Health Statistics (NCHS) nationwide multiple cause-of-death files with date of death through the U.S. EPA’s special arrangement with NCHS to support research (Centers for Disease Control and Prevention 2010). Using codes from the *International Classification of Diseases*, version 10 (ICD-10; World Health Organization 2007), we aggregated daily death counts to nonaccidental all-cause deaths (ICD-10, codes A00 through R99), cardiovascular deaths (ICD-10, codes I01 through I99), and respiratory deaths (ICD-10, codes J00 through J99).

### Meteorological data

We retrieved daily average temperature and dew point data for the Detroit Metro Airport and the Seattle-Tacoma International Airport from the National Oceanic and Atmospheric Administration, National Climatic Data Center (2009) and determined relative humidity with temperature and dew point. All daily mortality, pollutants, and weather data were processed using SAS software (version 9.1; SAS Institute Inc., Cary, NC).

### Methods

We used a Poisson regression model to examine short-term associations between ambient air pollution and mortality by cause. The regression model included an indicator for day of week, a smooth function of time with 8 degrees of freedom (df) per calendar year (4 df/year for season analyses) to control for seasonality and long-term trends (Peng et al. 2006), a smooth function of current-day temperature (3 df), a smooth function of delayed temperature (3 df), and a smooth function of humidity (3 df). For all of the smooth functions, we used a natural spline basis. We used consecutive number of study days (from 1 through 1,096 for 3 years) to fit natural cubic splines. We employed 3-day average lags (lag 1 to lag 3 days) for delayed temperature effects. The Poisson model also accommodated overdispersion. We examined 0-, 1-, 2-, and 3-day lag concentrations because previous studies with PM_2.5_ total mass showed little evidence of a strong association with health effects beyond 3 days (Dominici et al. 2006; Ostro et al. 2007; Peng et al. 2009). In presenting results, we computed excess risks for an interquartile range (IQR) increase of each pollutant. Because source impacts likely vary across seasons, we stratified the data set by warm (April–September) and cold (October–March) seasons.

We also employed a constrained (second-degree polynomial) distributed lag model using dlnm (version 1.2.4), an R package developed by Gasparrini et al. (2010), to estimate cumulative effect over multiple days. Because most of the significant associations in individual lag models occurred at lags of 0 through lag 2 days, we summed up the cumulative effects from lag 0 to lag 2 days in the distributed lag model. Because the risk estimates can change considerably depending on the model specifications (Schwartz 1994a, 1994b; Touloumi et al. 2004) in time-series models, we performed additional sensitivity analyses: use of alternative degrees of freedom (5 df/year and 12 df/year) for temporal adjustment and use of alternative degrees of freedom (4, 5, and 6 df over the temperature range) for smooth temperature terms. In addition, we also examined “other mortality” [all-cause minus (cardiovascular plus respiratory)] to check consistency of results with causal inference. All analyses were conducted using R (version 2.11.1; R Development Core Team 2010).

## Results

Tables 1 and 2 show the descriptive statistics of the ambient pollutants, daily death, and weather for Detroit and Seattle, respectively. In Detroit, PM_2.5_ and most components (Al, Fe, K, Ni, S, Si, V, Zn, and EC) had somewhat higher concentrations in the warm season. Na, CO, and NO_2_ levels were higher in the cold season. In Seattle, PM_2.5_, Fe, K, Zn, EC, CO, and NO_2_ showed higher levels in the cold season, whereas the levels of Al, Na, Ni, S, Si, and V were higher in the warm season.

The risk estimates at individual lag days often showed positive associations at multiple lag days, especially at lag 0 through lag 2 days. Thus, the results from the distributed lag models exhibited patterns of associations generally similar to those from the individual lag analysis. Therefore, for clarity, we present the results from the distributed lag models here but also describe some aspects of individual lag results [see Supplemental Material (doi:10.1289/ehp.1002613)] that were lost in the distributed lag results.

**Figure 1:**
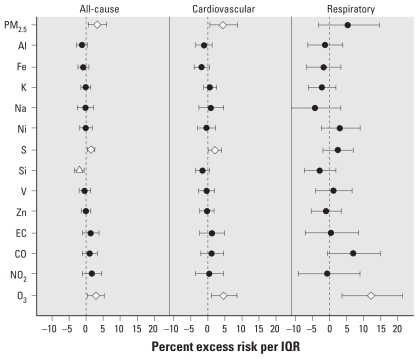
Percent excess mortality risk in the warm season in Detroit. The diamonds and triangle represent significantly (*p* < 0.05) positive and negative associations, respectively.

**Figure 2:**
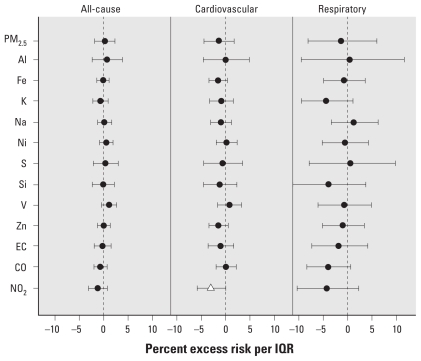
Percent excess mortality risk in the cold season in Detroit. The triangle represents significant (*p* < 0.05) associations.

**Figure 3:**
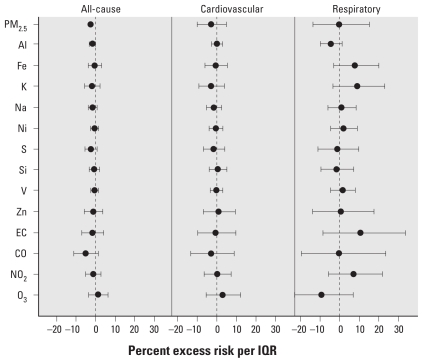
Percent excess mortality risk in the warm season in Seattle.

**Figure 4:**
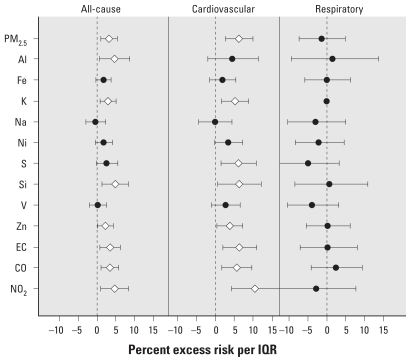
Percent excess mortality risk in the cold season in Seattle. The diamonds represent significant (*p* < 0.05) associations.

Figures 1–4 summarize the results of distributed lag model that produced the cumulative effects from lag 0 to 2 days. The most prominent contrast between the two cities was the seasonal pattern of the associations—generally, in Detroit, some pollutants were more positively associated with mortality outcomes in the warm season, whereas in Seattle, some pollutants showed stronger associations in the cold season.

In Detroit, we found significant positive associations for PM_2.5_, S, and O_3_ for all-cause and cardiovascular mortality in the warm season (Figure 1), suggesting a role of secondary pollutants. Si was significantly negatively associated with all-cause mortality. Only O_3_ was significantly associated with respiratory mortality in the warm season. We observed no significant association in the cold season, except for a significant negative association between NO_2_ and cardiovascular mortality (Figure 2). In the individual lag model results for Detroit [see Supplemental Material, Figures 1, 2 (doi:10.1289/ehp.1002613)], Ni, EC, CO, and NO_2_ showed significant positive associations at single-day lags.

In Seattle, in contrast to Detroit, we observed no significant association in the warm season for any mortality category (Figure 3), and multiple pollutants (PM_2.5_, Al, K, S, Si, Zn, EC, CO, and NO_2_) were significantly positively associated with all-cause or cardiovascular mortality in the cold season (Figure 4). We observed no significant association for respiratory mortality. In the individual lag model results [see Supplemental Material, Figures 3, 4 (doi:10.1289/ehp.1002613)], Al, Fe, Ni, (all at lag 1 day), and V (at lag 2 days) also showed positively significant associations in the cold season. In the warm season, O_3_ was significantly positively associated with all-cause mortality at lag 2 days. We observed inexplicable significantly negative associations at lag 0 day for PM_2.5_, Al, Fe, K, Na, Si, and CO for all-cause mortality in the warm season. Such negative associations might have been caused by overadjustment of the same-day temperature, and most of them disappeared when we removed the same-day temperature adjustment from the model as part of sensitivity analyses (data not shown).

The estimated risks were not sensitive to alternative degrees of freedom per year (5 df/year and 12 df/year vs. 8 df/year for the main analysis). We observed no indication of systematic difference in risk estimates [see Supplemental Material, Figures 5, 6 (doi:10.1289/ehp.1002613)]. Likewise, we examined the sensitivity of risk estimates to adjustment of degrees of freedom for temperature and delayed temperature, using 4, 5, and 6 df, as opposed to 3 df used in the original model, and found that the risk estimates were robust to the change in degrees of freedom to fit temperature effects (data not shown). The fitted temperature terms showed generally positive slopes for the same-day temperature and negative slopes for the average of 1- to 3-day-lag temperature for both cities for all-cause and cardiovascular deaths (data not shown).

In the analysis of “other cause” of deaths, although we observed a few significant associations (e.g., NO_2_ at lag 3 days in the cold season in Seattle, and at lag 1 day in the warm season in Detroit), the air pollutants’ associations with “other cause” deaths were, in general, much lower than those for cardiovascular and respiratory deaths (data not shown).

## Discussion

Our time-series analysis in two U.S. cities indicates considerable risk heterogeneity from pollutant to pollutant. This is consistent with the results of previous studies (Burnett et al. 2000; Mar et al. 2000; Metzger et al. 2004). Laden et al. (2000) examined source-oriented combinations of PM_2.5_ species from the Harvard Six Cities study and found that motor vehicle exhaust and coal combustion were associated with mortality, whereas the soil factor was not. In our all-year analysis, we found no association with Al; however, we observed significant associations with mortality during the cold season in Seattle. This is consistent with previous study results of Ostro et al. (2007) for PM_2.5_ and mortality in six California counties. We observed excess risks of 1.5–7.4% for cardiovascular and respiratory death in the Poisson regression model based on IQR increase of each pollutant, and fewer, yet larger, excess risks in the distributed lag model. In addition, the cardiovascular and respiratory mortality series exhibited different source and seasonal patterns in each city. The PM components and gaseous pollutants associated with mortality in Detroit appear most associated with warm-season secondary aerosols and traffic markers. In Seattle, the species associated with mortality include those for cold-season traffic and other combustion sources, such as residual oil and wood burning.

PM components have been examined as independent predictors in several epidemiological studies on local and regional scales, and the epidemiological evidence linking specific PM components to health risks is mixed (Anderson et al. 2001; Burnett et al. 1997; Chuang et al. 2007; Sarnat et al. 2006). Disparities in findings may result from the diversity of the study locations and their different pollutant concentrations and ratios, health outcomes, or the analytic methods. However, some studies (Laden et al. 2000; Metzger et al. 2004; Ostro et al. 2007, 2008; Tolbert et al. 2000; Zanobetti and Schwartz 2006) found associations between cardiovascular outcomes and EC, and the evidence of increased risk of cardiovascular mortality and EC reported in our studies is consistent with these previous findings. Potassium (K) is generally considered a reasonable marker for biomass combustion, including residential wood burning (Maykut et al. 2003; Watson et al. 2001), which is an important contributor to air pollution in Seattle. Our results showed a significant association between K and cardiovascular mortality, but only in Seattle, despite the fact that the concentration of K was higher in Detroit during the study period.

It is also noteworthy that the annual average PM_2.5_ concentration in Detroit (15.1 g/m^3^) is at the current National Ambient Air Quality Standard (NAAQS) (U.S. EPA 2010a), whereas the annual average PM_2.5_ concentration in Seattle (9.7 g/m^3^) would be in compliance with even the lowest concentration limit being considered as a revised annual average for PM_2.5_ (11–13 g/m^3^) (Samet 2010). Because cardiovascular mortality is considerably more closely associated with PM_2.5_ in Seattle than in Detroit, it suggests that PM_2.5_ pollution is associated with increases in daily mortality even at currently observed low levels (a mean of 9.7 g/m^3^) in Seattle, although further analyses are needed to shed light on the association between a low level of PM_2.5_ and health effects. The PM_2.5_ components and gaseous pollutants most closely associated with cardiovascular mortality in Seattle were K, S, Si, Zn, EC, NO_2_, and CO. Thus, reduction of cardiovascular mortality risk in Seattle may need to focus on residual oil combustion, the major source of S in this city; biomass burning, the major source of K; metal processing industries and incinerators, important sources of Zn; and motor vehicles, the major source of EC, NO_2_, and CO. The PM components and gaseous pollutants associated with daily mortality in Detroit (in the warm season only) were S and O_3_. Thus, reduction of cardiovascular and respiratory mortality risk in Detroit may need to focus most on traffic emissions and power plants, which are sources of sulfate and O_3_ precursors.

The mortality effects exhibited differing seasonal patterns in these two cities. In Detroit, significant associations between mortality and PM_2.5_ components and gaseous pollutants appeared only in the warm season, when there is more infiltration of outdoor air into occupied spaces. Detroit and Seattle showed similar temperature effects: Warm weather had positive associations with same-day health effects and negative associations with delayed health effects. However, the mortality showed opposite seasonal patterns in these cities, although the pollutant concentration variations did not indicate obvious seasonal patterns in either of the two cities. In Seattle, where the annual range of temperature was smaller, we observed significant associations between mortality and air pollutants in the cold season, when some pollutants had higher concentrations, for example K, EC, and CO, and others had lower concentrations, such as Al, S, and Si. Becker et al. (2005) similarly reported seasonal variation in the toxicity of PM, which they justified on the basis that PM in different seasons contains different elemental compositions that are affected by changes of local environment and weather conditions.

Air-conditioned buildings may keep people indoors from being exposed to high levels of outdoor air pollutants. According to the American Housing Survey ([Bibr b45-ehp-119-461][Bibr b46-ehp-119-461]), the percentages of housing with air conditioning were 84.4% in Detroit in 2003, and 14.7% in Seattle in 2004. Homes in Seattle had a much lower percentage of air conditioning than did those in Detroit, which may have been due to the milder weather in Seattle. In our case, the average temperatures in the warm and cold seasons were 64.4ºF and 35.7ºF in Detroit and 59.6ºF and 45ºF in Seattle, respectively. Many air pollutants are highly correlated spatially across a metropolitan area, but they may not permeate equally in all buildings (Wechsler et al. 1989). High percentages of air-conditioned homes and buildings may reduce the average ability of air pollutants to reach residents for much of the day, which may diminish the overall population adverse health implications of outdoor air pollution in those cities versus cities without extensive air conditioning. As a result, it is likely that, in cities that have extensive use of air conditioning, the estimated health effects on exposed individuals may be underestimated compared with effects estimated in communities having milder climates and more limited use of air conditioning. The influence of such potential effect modifiers needs further investigation.

It is important to note the limitations of our study. First, the use of a single location in each city to represent the concentrations of PM_2.5_ components is likely to lead to random measurement error and the potential for downwardly biased effect estimates that may vary with PM source. Therefore, one of our concerns is a monitor’s representation of regional, subregional, and local air pollution exposures for the population in these two metropolitan areas. Some investigators have also questioned whether the observed associations are plausible given these findings. However, Schwartz et al. (1996) noted that daily mortality is calculated over the population, and the relevant exposure measure is the mean of personal exposures on that day, which is probably more tightly correlated with central station monitoring of outdoor air pollution than with individual exposures. A previous study (Mage et al. 1999) also indicated that such data from central monitoring sites are usually, although not always, highly correlated with the average of individual exposures in a population. We should also note that an application of such time-series epidemiological analyses is often to help set ambient standards that will ultimately be monitored at a central site, and from a regulatory perspective, the central site ambient pollution levels are most relevant.

Another limitation on the interpretation of our results is that our definitions of cardiovascular and respiratory mortalities reduce the specificity of the estimated associations, compared with more narrowly defined categories. However, the countervailing benefit of this approach is the increased power to detect associations between PM components and health outcomes. As in many other studies, we had to strike a balance between statistical power and specificity of the outcome. A third limitation is that we conducted this research for only two cities with daily speciation data. More states should follow these two states’ lead in retaining daily sample filters to allow distributed lag modeling and to improve the power of future analyses.

Our results add to those previously reported in two important ways. First, in our study, with 3 years of daily PM_2.5_ speciation data for Detroit and Seattle, we were able to examine the distribution of mortality effects over time, characterize the short-term health effects of PM_2.5_ more thoroughly, and potentially provide insights for better targeted regulation to reduce source emissions. Second, this study indicates sources of PM air pollution that could be targeted as part of a comprehensive air quality control strategy. Ambient PM_2.5_ is produced by numerous emission sources, and individual PM_2.5_ components identified as having associations with health outcomes may be acting as markers for other components or a set of components with similar sources. For example, EC results from combustion of fossil fuels, but also from the combustion of biomass and from industry (HEI 1995). Our results showed that PM_2.5_ components and gaseous pollutants associated with cardiovascular mortality in Detroit appear to be associated with secondary aerosols and traffic markers in the warm season, whereas in Seattle, the PM components and gaseous pollutants associated with cardiovascular mortality include traffic and other combustion sources in the cold season, such as residual oil burning, incinerators, and wood smoke.

## Conclusion

In summary, we analyzed data from Detroit and Seattle to assess the associations between daily mortality and PM_2.5_ and its elemental components, as well as gaseous pollutants. We found some inconsistent results: although we observed slightly more daily deaths in the cold season than in the warm season in both cities, the daily mortality associations in these cities showed opposite seasonal patterns, whereas the pollutants did not indicate obvious seasonal concentration patterns in either city. It is generally accepted that weather conditions affect mortality rates, and it is difficult to rule out the possibility that there are other causes of mortality. Our results were quite different depending on the location, so we could not draw a definitive conclusion as to whether this is due to differences in the composition of PM_2.5_ and other air pollutants, differences in weather, or some other variables.

Most important, our study found a major contrast in seasonal pattern of association between air pollutants and daily mortality in two major U.S. cities. Along with the previous works of others, our results provide substantial evidence that a more refined ambient air quality control strategy, based on the effects of PM_2.5_ components, can and should be developed. The range of chemical components and sources linked to various health responses supports the hypothesis that the toxicity of various components may vary. Although some sources, such as vehicular traffic and power plants, have been indicated as important contributors in this and other published analyses, understanding the health effect contributions of various pollutants and their sources still needs further investigation in more cities with daily speciation data.

## Figures and Tables

**Table 1:** Descriptive statistics of Detroit data.

Variable	Minimum	Median	Maximum	IQR	Mean (warm)	Mean (cold)	SD
PM_2.5_ (μg/m^3^)	2.2000	13.2000	65.8000	11.0000	15.2562	14.8750	8.7793
PM_2.5_ components (μg/m^3^)							
Al	0.0000	0.0095	0.3407	0.0203	0.0207	0.0085	0.0213
Fe	0.0129	0.0751	0.7641	0.0697	0.1090	0.0847	0.0775
K	0.0052	0.0501	0.7434	0.0337	0.0664	0.0533	0.0473
Na	0.0000	0.0421	0.4121	0.0401	0.0415	0.0615	0.0423
Ni	0.0000	0.0003	0.0096	0.0007	0.0006	0.0004	0.0007
S	0.1466	0.8786	6.9578	0.9262	1.5377	0.8904	1.0450
Si	0.0000	0.0451	0.7358	0.0382	0.0703	0.0410	0.0480
V	0.0000	0.0005	0.0172	0.0016	0.0014	0.0008	0.0016
Zn	0.0022	0.0190	0.2693	0.0192	0.0264	0.0242	0.0229
EC	0.1197	0.7090	3.2770	0.4250	0.8246	0.7210	0.3601
CO (ppm)	0.0000	0.2875	1.8000	0.1875	0.3012	0.3403	0.1838
NO_2_ (ppm)	0.0005	0.0167	0.0810	0.0099	0.0149	0.0203	0.0083
O_3_ (ppm)^a^	0.0023	0.0251	0.0626	0.0123	0.0259	NA	0.0100
All-cause mortality^b^	58.0000	93.0000	135.0000	16.0000	88.1913	97.8611	11.9840
Cardiovascular mortality^b^	19.0000	39.0000	67.0000	9.2500	37.5519	42.4442	7.2971
Respiratory mortality^b^	0.0000	8.0000	20.0000	4.0000	7.3661	8.8483	3.1271
Temperature (ºF)	3.8000	50.7000	85.6000	32.4250	64.4377	35.6728	18.7186
Dew point (ºF)	−7.1000	41.6500	74.0000	29.6000	53.6987	27.7530	18.2115
Relative humidity (%)	31.4000	75.6000	98.9000	15.8000	70.1701	77.9995	12.0134

NA, not available.

a O_3_ data for cold season were not available.

b Deaths/day.

**Table 2:** Descriptive statistics of Seattle data.

Variable	Minimum	Median	Maximum	IQR	Mean (warm)	Mean (cold)	SD
PM_2.5_ (μg/m^3^)	0.8000	7.9000	41.3000	7.4000	7.9877	11.4144	6.3684
PM_2.5_ components (μg/m^3^)							
Al	0.0000	0.0064	0.2445	0.0163	0.0159	0.0067	0.0185
Fe	0.0035	0.0473	0.4043	0.0462	0.0608	0.0615	0.0466
K	0.0072	0.0388	0.2253	0.0300	0.0388	0.0546	0.0287
Na	0.0000	0.1011	0.9914	0.1241	0.1580	0.1132	0.1227
Ni	0.0000	0.0013	0.0313	0.0023	0.0028	0.0019	0.0029
S	0.0431	0.3967	1.9252	0.3443	0.5122	0.3714	0.2794
Si	0.0000	0.0281	0.6552	0.0364	0.0507	0.0278	0.0425
V	0.0000	0.0019	0.0581	0.0051	0.0054	0.0031	0.0065
Zn	0.0006	0.0088	0.0530	0.0081	0.0088	0.0133	0.0076
EC	0.0260	0.5251	2.3368	0.5072	0.4958	0.7767	0.3918
CO (ppm)	0.1000	1.1000	4.8000	0.7000	0.9042	1.5121	0.5958
NO_2_ (ppm)	0.0030	0.0189	0.0452	0.0105	0.0178	0.0210	0.0074
O_3_ (ppm)^a^	0.0027	0.0199	0.0400	0.0097	0.0202	NA	0.0072
All-cause mortality^b^	20.0000	38.0000	61.0000	9.0000	36.1985	39.8940	6.6830
Cardiovascular mortality^b^	5.0000	14.0000	31.0000	6.0000	13.2587	14.7075	3.9741
Respiratory mortality^b^	0.0000	4.0000	12.0000	3.0000	3.4572	4.3821	2.0862
Temperature (ºF)	22.9000	51.2000	82.7000	15.6000	59.5860	45.0408	10.0539
Dew point (ºF)	4.2000	44.2000	62.8000	12.2000	49.0308	39.2920	8.4259
Relative humidity (%)	7.5000	80.6000	100.0000	18.9000	70.6807	84.0314	15.1027

NA, not available.

a O_3_ data for cold season were not available.

b Deaths/day.
